# Supplementary material for: Heat adaptation of phage T7 under an extended genetic code
Source: Virus Evol. 2021 Dec 1;7(2):veab100. doi: 10.1093/ve/veab100 (PMC8923235; doi:10.1093/ve/veab100)
Supplement: veab100_Supp [file veab100_supp.zip › Sequencing_Analysis.zip!/Sequencing_Analysis/A2/evidence/SNP_9.html]

BRESEQ :: Evidence


| Predicted mutation | | | | | | |
| --- | --- | --- | --- | --- | --- | --- |
| evidence | seq id | position | mutation | annotation | gene | description |
| RA | NC\_001604.1 | 27,932 | T→C | intergenic (–/–) | – / – | –/– |

| Read alignment evidence... | | | | | | | | | | | |
| --- | --- | --- | --- | --- | --- | --- | --- | --- | --- | --- | --- |
|  | seq id | position | | ref | new | freq | score (cons/poly) | reads | annotation | genes | product |
| \* | NC\_001604.1 | 27,932 | 0 | T | C | 85.4% | 1111.3 / 93.7 | 502 | intergenic (–/–) | *–/–* | –/– |
| Reads supporting (aligned to +/- strand):  **ref** base T (32/39);  **new** base C (186/228);  **total** (222/280) | | | | | | | | | | | |
| Fisher's exact test for biased strand distribution *p*-value = 1.00e+00 | | | | | | | | | | | |
| Kolmogorov-Smirnov test that lower quality scores support variant *p*-value = 1.00e+00 | | | | | | | | | | | |

Only 100 of 503 total aligned reads displayed.  

`CCTCAAGACTATCGGTGCGGTATTCCATGAAGAGTACACACGAGATGGTCAATTTCAGTTATTTACAATCACGAAAGGAGGATAACCATATGTGTTGGGCAGCCGCAATACCTATCGCTATATCTGGCGCTCAGGCTATCAGTGGTCAGAACGCTCAGGCCAAAATGATTGCCGCTCAGACCGCTGCTGGTCGTCGTCAAGCTATGGAAATCATGAGGCAGACGAACATCCAGAATGCTGACCTATCGTTGCAAGCTCGAAGTAAACTTGAGGAAGCGTCCGCCGAGTTGACCTCACAGAACATGCAGAAGGTCCAAGCTATTGGGTCTATCCGAGCGGCTATCGGAGAGAGTATGCTTGAAGGTTCCTCAATGGACCGCATTAAGCGAGTCACAGAAGGACAGTTCATTCGGGAAGCCAATATGGTAACTGAGAACTATCGCCGTGACTACCAAGCAATCTTCGCACAGCAACTTGGTGGTACTCAAAGTGCTGCAAGTCAGATTGACGAAATCTATAAGAGCGAACAGAAACAGAAGAGTAAGCTACAGATGGTTCTGGACCCACTGGCTATCATGGGGTCTTCCGCTG  >  NC_001604.1/27639‑28229`  

                                                                                                                                                                                                                                                                                                     `|`                                                                                                                                                                                                                                                                                                           
   `cAAGACTATCGGTGCGGTATTCCATGAAGAGTACACACGAGATGGTCAATTTCAGTTATTTACAATCACGAAAGGAGGATAACCATATGTGTTGGGCAGCCGCAATACCTATCGCTATATCTGGCGCTCAGGCTATCAGTGATCAGAACGCTCAGGCCAAAATGATTGCCGCTCAGACCGCTGCTGGTCGTCGTCAAGCTATGGAAATCATGAGGCAGACGAACATCCAGAATGCTGACCTATCGTTGCAAGCTCGAAGTAAACTTGAGGAAGCGTCCGCCGAGTTGACCCCACAGAACAt                                                                                                                                                                                                                                                                                                 >  1:27343/1‑301 (MQ=255)`  
     `aGACTATCGGTGCGGTATTCCATGAAGAGTACACACGAGATGGTCAATTTCAGTTATTTACAATCACGAAAGGAGGATAACCATATGTGTTGGGCAGCCGCAATACCTATCGCTATATCTGGCGCTCAGGCTATCAGTGATCAGAACGCTCAGGCCAAAATGATTGCCGCTCAGACCGCTGCTGGTCGTCGTCAAGCTATGGAAATCATGAGGCAGACGAACATCCAGAATGCTGACCTATCGTTGCAAGCTCGAAGTAAACTTGAGGAAGCGTCCGCCGAGTTGACCCCACAGAACATGc                                                                                                                                                                                                                                                                                               >  1:450384/1‑301 (MQ=255)`  
      `ggtattccatgaagagtacacacgagatggtcaatttcagttatttacaatcacgaaaggaggataaccatatgtgttgggcagccgcaatacctatcgctatatctggCGCTATATCTGGCGCTCAGGCTATCAGTGGTCAGAACGCTCAGGCCAAAATGATTGCCGCTCAGACCGCTGCTGGTCGTCGTCAAGCTATGGAAATCATGAGGCAGACGAACATCCAGAATGCTGACCTATCGTTGCAAGCTCGAAGTAAACTTGAGGAAGCGTCCGCCGAGTTGACGTCACAGAACATGCa                                                                                                                                                                                                                                                                                              >  1:752513‑M2/110‑301 (MQ=255)`  
        `tgtgtataagagacagccATGAAGAGTACACACGAGATGGTCAATTTCAGTTATTTACAATCACGAAAGGAGGATAACCATATGTGTTGGGCAGCCGCAATACCTATCGCTATATCTGGCGCTCAGGCTATCAGTGATCAGAACGCTCAGGCCAAAATGATTGTCGATCAGACCGCTGCTGGTCGTCGTCAAGCTATGGAAATCATGAGGCAGACGAACATCCAGAATGCTGACCTATCGTTGCAAGCTCGAAGTAAACTTGAGGAAGCGTCCGCCGAGTTGACCACACAGAACATGCAGa                                                                                                                                                                                                                                                                                            <  1:331315/285‑1 (MQ=255)`  
         `attccatgaagagtacacacgagatggtaaatttcagttatttacaatcacgaaaggaggataaccatatgtgttgggcagccgcaatacctatcgctatatctggCGCTATATCTGGCGCTCAGGCTATCAGTGGTCAGAACGCTCAGGCCAAAATGATTGCCGCTCAGACCGCTGCTGGTCGTCGTCAAGCTATGGAAATCATGAGGCAGACGAACATCCAGAATGCTGACCTATCGTTGAAAGCTCGTAGTAAACTTGAGGAAGCGTCCGCCGAGTTGACCCCACAGAACATGCAGaa                                                                                                                                                                                                                                                                                           >  1:210940‑M2/107‑301 (MQ=255)`  
            `ccatgaagagtacacaagagatggtcaatttcagttatttacaatcacgaaaggaggataaccatatgtgttgggcagccgcaatacctatcgctatatctggCGCTATATCTGGCGCTCAGGCTATCAGTGATCAGAACGCTCAGGCCAAAATGATTGCCGCTCAGACCGCTGCTGGTCGTCGTCAAGCTATGGAAATCATGAGGCAGACGAACATCCAGAATGCTGACCTATCGTTGCAAGCTCGAAGTAAACTTGAGGAAGCGTCCGCCGAGTTGACCCCACAGAACATGCAGAAGGt                                                                                                                                                                                                                                                                                        >  1:484651‑M2/104‑301 (MQ=255)`  
             `catgaagagtacacacgagatggtcaatttcagttatttacaatcacgaaaggaggataaccatatgtgttgggcagccgcaatacctatcgctatatctggCGCTATATCTGGCGCTCAGGCTATCAGTGGTCAGAACGCTCAGGCCAAAATGATTGCCGCTCAGACCGCTGCTGGTCGTCGTCAAGCTATGGAAATCATTAGGCAGACGAACATCCAGAATGCTGACCTATCGTTGCAAGCTCGAAGTAAACTTGAGGAAGCGTCCGCCGAGTTGACCCCACAGAACATGCAGAAGGTc                                                                                                                                                                                                                                                                                       <  1:507115‑M2/199‑1 (MQ=255)`  
                  `ttataagagacaggagTACACACGAGATGGTCAATTTCAGTTATTTACAATCACGAAAGGAGGATAACCATATGTGTTGGGCAGCCGCAATACCTATCGCTATATCTGGCGCTCAGGCTATCAGTGATCAGAACGCTCAGGCCAAAATGATTGCCGCTCAGACCGCTGCTGGTCGTCGTCAAGCTATGGAAATCATGAGGCAGACGAACATCCAGAATGCTGACATATCGTTGCAAGCTCGAAGTAAACTTGAGGAAGCGTCCGCCGAGTTGACCCCACAGAACATGCAGAAGGTCCagct                                                                                                                                                                                                                                                                                  <  1:347908/288‑4 (MQ=255)`  
                  `caggtacacacgagatggtcaatttcaggtatttacaatcacgaaaggaggataaccatatgtgttgggcagccgcaatacctatcgctatatctggCGCTATATCTGGCGCTCAGGCTATCAGTGGTCAGAACGCTCAGGCCAAAATGATTGCCGCTCAGACCGCTGCTGGTCGTCGTCAAGCTATGGAAATCATGAGGCAGACGAACATCCAGAATGCTGACCTATCGTTGCAAGCTCGAAGTAAACTTGAGGAAGCGTCCGCCGAGTTGACCTCACAGAACATGCAGAAGGTCCAAGc                                                                                                                                                                                                                                                                                  <  1:67217‑M2/204‑1 (MQ=255)`  
                   `gagtacacacgagatggtcaatttcagttatttacaatcacgaaaggaggataaccatatgtgttgggcagccgcaatacctatcgctatatctggCGCTATATCTGGCGCTCAGGCTATCAGTGGTCAGAACGCTCAGGCCAAAATGATTGCCGCTCAGACCGCTGCTGGTCGTCGTCAAGCTATGGAAATCATGAGGCAGACGAACATCCAGAATGCTGACCTATCGTTGCAAGCTCGAAGTAAACTTGAGGAAGCGTCCGCCGAGTTGACCTCACAGAACATGCAGAAGGTCCAAGCt                                                                                                                                                                                                                                                                                 <  1:292291‑M2/205‑1 (MQ=255)`  
                   `gTATTCCATGAAGAGTACACACGAGATGGTCAATTTCAGTTATTTACAATCACGAAAGGAGGATAACCATATGTGTTGGGCAGCCGCAATACCTATCGCTATATCTGGCGCTCAGGCTATCAGTGATCAGAACGCTCAGGCCAAAATGATTGCCGCTCAGACCGCTGCTGGTCGTCGTCAAGCTATGGAAATCATGAGGCAGACGAACATCCAGAATGCTGACCTATCGTTGCAAGCTAGAAGTAAACTTGAGGAAGCGTCCGCCGAGTTGACCCCACAGAACATGCAGAAGGTctgtctc                                                                                                                                                                                                                                                                                 >  1:267035/1‑295 (MQ=255)`  
                     `gtacacacgagatggtcaatttcagctatttacaatcacaaaaggaggataaccatatgcgttgggcagccgcaatacatatcgctatatctggCGCTATATCTGGCGCTCAGGCTATTAGCGGTCATAACGCTCAGTCCAAAATGTTTGCCCCTCAGACCGCTGCTGGTCGTCGTCAAGCTATGGAAATCATGCGGAAGACTAACATTCAGAACGCTCACCTATCGTTGCAAGATCGAAGTAAACTTGAGGAAGCGTCCGCCGAGTTGACCCCACAGAACCTGCAGAAGGTCCACGCTAt                                                                                                                                                                                                                                                                               >  1:679148‑M2/95‑301 (MQ=255)`  
                     `aTTCCATGAAGAGTACACACGAGATGGTCAATTTCAGTTATTTACAATCACTAAAGGATGATAACCATATGTGTTGGGCAGCTGCAATACCTATCTCTATATCTGGCGCTCAGGCTATCAGTGATCAGAACGCTCAGACCAAAATGATTGCCGCTCAGACCGCTGCTGGTCGTCGTCAAGCAATGGAAATCATGAGGCAGACGAACATCCAGAATGCTGACCTATCGTTGAAAGCGAGAAGGACACTTGAGGAAGCGTCCGCAGAGGTGACCCCACAGAACATGGAAAAGGTACAAGATag                                                                                                                                                                                                                                                                               >  1:319202/1‑300 (MQ=255)`  
                     `aTTCCATGAAGAGTACACACGAGATGGTCAATTTCAGTTATTTACAATCACGAAAGGAGGATAACCATATGTGTTGGGCAGCCGCAATACCTATCGCTATATCTGGCGCTCAGGCTATCAGTGATCAGAACGCTCAGGCCAAAATGATTGCCGCTCAGACCGCTGCTGGTCGTCGTCAAGCTATGGAAATCATGAGGCAGACGAACATCCAGAATGCTGACCTATCGTTGCAAGCTCGAAGTAAACTTGAGGAAGCGTCCACCGCGTTGACCCCACAGAACATGCAGAAGGTCCAAGCTAt                                                                                                                                                                                                                                                                               >  1:800809/1‑301 (MQ=255)`  
                      `tacacacgagatggtcaatttcagttatttacaatcacgaaaggaggataaccatatgtgttgggcagccgcaatacctatcgctatatctggCGCTATATCTGGCGCTCAGGCTATCAGTGATCAGAACGCTCAGGCCAAAATGATTGCCGCTCAGACCGCTGCTGGTCGTCGTCAAGCTATGGAAATCATGAGGCAGACGAACATCCAGAATGCTGACCTATCGTTGCAAGCTCGAAGTAAACTTGAGGAAGCGTCCGCCGAGTTGACCCCACAGAACATGCAGAAGGTCCAAGCTAtt                                                                                                                                                                                                                                                                              >  1:708590‑M2/94‑301 (MQ=255)`  
                       `tCCATGAAGAGTACACACGAGATGGTCAATTTCAGTTATTTACAATCACGAAATGAGGATAACCATATGTGTTGGGCAGCCGCAATACCTATCGCTATATCTGGCGCTCAGGCTATCAGTGATCAGAACGCTCAGGCCAAAATGATTGCCGCTCAGACCGCTGCTGGTCGTCGTAAAGCTATGGAAAGCATGAGGCATACGAACATACAGAATGCTGACCTATCGTTGCAAGCTCGAAGTAAACTTGAGGAAGCGTACGTCGAGTCGACCTGACAGAACATGCAGAAGGACCAAGCTATTg                                                                                                                                                                                                                                                                             >  1:357175/1‑301 (MQ=255)`  
                       `tCCATGAAGAGTACACACGAGATGGTCAATTTCAGTTATTTACAATCACGAAAGGATGATAACCATATGTGTTGGGCAGCCGCAATACCTATCGCTATATCTGGCGCTCAGGCTATCAGTGATCAGAACGCTCAGGCCAAAATGATTGCCGCTCAGACCGCTGCTGGTCGTCGTCAAGCTATGGAAATCATGAGGCAGACGAACATCCAGAATGCTGACCTATCGTTGCAAGCTCGAAGTAAACTTGAGGAAGCGTCCGCCGAGTTGACCCCACAGAACATGCAGAATGTCCAAGCTATTg                                                                                                                                                                                                                                                                             >  1:153709/1‑301 (MQ=255)`  
                       `tCCATGAAGAGTACACACGAGATGGTCAATTTCAGTTATTTACAATCACGAAAGGAGGATAACCATATGTGTTGGGCAGCCGCAATACCTATCGCTATATCTGGCGCTCAGGCTATCAGTGATCAGAACGCTCAGGCCAAAATGATTGCCGCTCAGACCGCTGCTGGTCGTCGTCAAGCTATGGAAATCATGAGGCAGACGAACATCCAGAATGCTGACCTATCGTTGCAAGCTCGAAGTAAACTTGAGGAAGCGTCCGCCGAGTTGACCCCACAGAACATGCAGAAGGTCCAAGCTATTg                                                                                                                                                                                                                                                                             >  1:384860/1‑301 (MQ=255)`  
                       `acacacgagatggtcaatttcagttatttacaatcacgaaaggaggataaccatatgtgttgggcagccgcaatacctatcgctatatctggCGCTATATCTGGCGCTCAGGCTATCAGTGGTCAGAACGCTCAGGCCAAAATGATTGCCGCTCAGACCGCTGCTGGTCGTCGTCAAGCTATGGAAATCATGAGGCAGACGAACATCCAGAATGCTGACCTATCGTTGCAATCTCGAAGTAAACTTGAGGAAGCGTCCGCCGAGTTGACCCCACAGAACATGCAGAAGGTCCAAGCTATTg                                                                                                                                                                                                                                                                             >  1:311853‑M2/93‑301 (MQ=255)`  
                         `cATGAAGAGTACACACGAGATGGTCAATTTCAGTTATTTACAATCACGAAAGGAGGATAACCATATGTGTTGGGCAGCCGCAATACCTATCGCTATATCTGGCGCTCAGGCTATCAGTGATCAGAACGCTCAGGCCAAAATGATTGTCGCTCAGACCGCTGCTGGTCGTCGTCAAGCTATGGAAATCATGAGGCAGACGAACATCCAGAATGCTGACCTATCGTTGCAAGCTCGAAGTAAACTTGAGGAAGCGTACGCCGAGTTGACCCCACAGAACATGCAGAAGGTCCAAGCTATTggg                                                                                                                                                                                                                                                                           <  1:771181/301‑1 (MQ=255)`  
                         `aaTGAAGAGTACACACGAGATGGTCAATTTCAGTTATTTACAATCACGAAAGGAGGATAACCATATGTGTTGGGCAGCCGCAATACCTATCGCTATATCTGGCGCTCAGGCTATCAGTGATCAGAACGCTCAGGCCAAAATGATTGCCGCTCAGACCGCTGCTGGTCGTCGTCAAGCTATGGAAATCATGAGGCAGACGAACATCCAGAATGCTGACCTATCGTTGCAAGCTCGAAGTAAACTTGAGGAAGCGTCCGCCGAGTTGACCCCACAGAACATGCAGAAGGTCCAAGCTATTggg                                                                                                                                                                                                                                                                           <  1:650497/300‑1 (MQ=255)`  
                          `aTGAAGAGTACACACGAGATGGTCAATTTCAGTTATTTACAATCACGAAAGGAGGATAACCATATGTGTTGGGCAGCCGCAATACCTATCGCTATATCTGGCGCTCAGGCTATCAGTGATCAGAACGCTCAGGCCAAAATGATTGCCGCTCAGACCGCTGCTGGTCGTCGTCAAGCTATGGAAATCATGAGGCAGACGAACATCCAGAATGCTGACCTATCGTTGCAAGCTCGAAGTAAACTTGAGGAAGCGTCCGCCGAGTTGACCTCACAGAACATGCAGAAGGTCCAAGCTATTGGGt                                                                                                                                                                                                                                                                          <  1:425044/301‑1 (MQ=255)`  
                           `agacaggTACACACGAGATGGTCAATTTCAGTTATTTACAATCACGAAAGGAGGATAACCATATGTGTTGGGCAGCCGCAATACCTATCGCTATATCTGGCGCTCAGGCTATCAGTGATCAGAACGCTCAGGCCAAAATGATTGCCGCTCAGACCGCTGCTGGTCGTCGTCAAGCTATGGAAATCATGAGGCAGACGAACATCCAGAATGCTGACCTATCGTTGCAAGCTCGAAGTAAACTTGAGGAAGCGTCCGCCGAGTTGACCCCACAGAACATGCAGAAGGTCCAAGCTATTGGGTc                                                                                                                                                                                                                                                                         <  1:82611/295‑1 (MQ=255)`  
                              `agagTACACACGAGATGGTCAATTTAAGTTATTTACAATCACGAAAGGAGGATAACCATATGTGTTGGGCAGCCGCAATACCTATCGCTATATCTGGCGCTCAGGCTATCAGTGATCAGAACGCTCAGGCAAAAATGATTGCCGCTCAGACCGCTGCTGGTCGTCGTCAAGGTATGGAAATCATGAGGCAGACGAACATCCAGAATGCTGACCTATCGTTGCAAGCTCGAAGTAAACTTGAGGAAGCGTCCGCCGAGTTGACCACACAGAACATGCAGAAGGTCCAAGCtctgtctcttat                                                                                                                                                                                                                                                                      >  1:395348/1‑290 (MQ=255)`  
                               `gtgtataagagacagggTCAATTTCAGTTATTTACAATCACGAAAGGAGGATAACCATATGTGTTGGGCAGCCGCAATACCTATCGCTATATCTGGCGCTCAGGCTATCAGTGATCAGAACGCTCAGGCCAAAATGATTGCCGCTCAGACCGCTGCTGGTCGTCGTCAAGCTATGGAAATCATGAGGCAGACGAACATCCAGAATGCTGACCTATCGTTGCAAGCTCGAAGTAAACTTGAGGAAGCGTCCGCCGAGTTGACCCCACAGAACATGCAGAAGGTCCAAGCTATTGGGTCTATc                                                                                                                                                                                                                                                                     <  1:599265/286‑1 (MQ=255)`  
                               `gagTACACACGATATGGTCAATTTCAGTTATTTACAATCACGAAAGGAGGATAACCATATGTGTTGGGCAGCTGCAATACCTATCGCTATATCTGGCGCTCAGGCTATCAGTGATCAGAACGCTCAGGCCAAAATGATTGCCGCTCAGACCGCTGCTGGTCGTCGTCAAGCTATGGAAATCATGAGGCAGACGAACATCCAGAATGCTGACCTATCGTTGCAAGCTCGAAGTAAACTTGAGGAAGCGTCCGCCGAGTTGACCCCACAGAACATGCAGAAGGTCCAAGCTATTGGGTCTATc                                                                                                                                                                                                                                                                     <  1:125632/301‑1 (MQ=255)`  
                               `gagTACACACGAGATGGTCAATTTCAGTTATTTACAATCACGAAAGGAGGATAACCTTATGTGTTTGGCAGCCGCAATACCTATCGCTATATCTGGCGCTCAGGCTATCAGTGATCAGAACGCTCAGGCCAAAATGATTGCCGCTCAGACCGCTGCTGGTCGTCGTCAAGCTATGGAAATCATGAGGCAGACGAACATCCAGAATGCTGACCTATCGTTGCAAGCTCGAAGTAAACTTGAGGAAGCGTCCGCTGAGTTGACCCCACAGAACATGCAGAAGGTCCAAGCTATTGGGTCTATc                                                                                                                                                                                                                                                                     >  1:581949/1‑301 (MQ=255)`  
                               `gagTACACACGAGATGGTCAATTTCAGTTATTTACAATCACGAAAGGAGGATAACCATATGTGTTGGGCAGCCGCAATACCTATCGCTATATCTGGCGCTCAGGCTATCAGTGATCAGAACGCTCAGGCCAAAATGATTGCCGCTCAGACCGCTGCTGGTCGTCGTCAAGCTATGGAAATCATGAGGCAGACGAACATCCAGAATGCTGACCTATCGTTGCAAGCTCGAAGTAAACTTGCTGACGCGTCCGCCGAGTTGACCCCACAGAACCTGCCGAAGGTcctgtctcttatcaacatc                                                                                                                                                                                                                                                                     >  1:807654/1‑284 (MQ=255)`  
                                 `gTACACACGAGATGGTCAATTTCAGTTATTTACAATCACGAAAGGATGATAACCATATGTGTTGGGCAGCCGCAATACCTATCGCTATATCTGGCGCTCAGGCTATCAGTGATCAGAACGCTCAGGCCAAAATGATTGCCGCTCAGACCGCTGCTGGTCGTCGTCAAGCTATGGAAATCATGAGGCAGACGAACATCCAGAATGCTGACCTATCGTTGCAAGCTCGAAGTAAACTTGAGGAAGCGTCCGCCGAGTTGACCCCACAGAACATGCAGAAGGTCCAAGCTATTGGGTCTATCCg                                                                                                                                                                                                                                                                   <  1:727973/301‑1 (MQ=255)`  
                                 `gTACACACGAGATGGTCAATTTCAGTTATTTACAATCACGAAAGGAGGATAACCATATGTGTTGGGCAGCCGCAATACCTATCGCTATATCTGGCGCTCAGGCTATCAGTTATCAGAACGCTCAGGCCAAAATGATTGCCGCTCAGACCGCTGCTGGTCGTCGTCAAGCTATGGAAATCATGAGGCAGACGAACATCCAGAATGCTGACCTATCGTTGCAAGCTCGAAGTAAACTTGAGGAAGCGTCCGCCGAGTTGACCCCACAGCACATGCAGAAGGTCCAAGCGATTGGGTCTATCCg                                                                                                                                                                                                                                                                   >  1:254602/1‑301 (MQ=255)`  
                                 `gTACACACGAGATGGTCAATTTCAGTTATTTACAATCACGAAAGGAGGATAACCATATGTGTTGGGCAGCCGCAATACCTATCGCTATATCTGGCGCTCAGGCTATCAGTGATCAGAACGCTCAGGCCAAAATGATTGCCGCTCAGACCGCTGCTGGTCGTCGTCAAGCTATGGAAATCATGAGGCAGACGAACATCCAGAATGCTGACCTATCGTTGCAAGCTCGAAGTAAACTTGAGGAAGCGTCCGCCGAGTTGACCCCACAGAACATGCAGAAGGTCCAAGCTATTGGGTCTATCCg                                                                                                                                                                                                                                                                   <  1:610199/301‑1 (MQ=255)`  
                                 `gTACACACGAGATGGTCAATTTCAGTTATTTACAATCACGAAAGGAGGATAACCATATGTGTTGGGCAGCCGCAATACCTATCGCTATATCTGGCGCTCAGGCTATCAGTGATCAGAACGCTCAGGCCAAAATGATTGCCGCTCAGACCGCTGCTGGTCGTCGTCAAGCTATGGAAATCATGAGGCAGACGAACATCCAGAATGCTGACATATCGTTGCAAGCTCGAAGTAAACTTGAGGAAGCGTCCGCCGAGTTGACCCCACAGAACATGCAGACGGTCCAAGCTATTGGGTCTATcct                                                                                                                                                                                                                                                                   >  1:158359/1‑300 (MQ=255)`  
                                 `gTACACACGAGATGGTCAATTTCAGTTATTTACAATCACGAAAGGAGGATAACCATATGTGTTGGGCAGCCGCAATACCTATCGCTATATCTGGCGCTCAGGCTATCAGTGATAAGAACGCTCAGGCCAAAATGATTGCCGCTCAGACCGCTGCTGGTCGTCGTCAAGCTATGGAAATCATGAGGCAGACGAACATCCAGAATGCTGACCTATCGTTGCAAGCTCGAAGTAAACTTGAGGAAGCGTCCGCCGAGTTGACCCCACAGAACATGCAGAAGGTCCAAGCTATTGGGTCTATCCg                                                                                                                                                                                                                                                                   <  1:474149/301‑1 (MQ=255)`  
                                     `acacGATATGGTCAATTTCAATTATTTACAATCACGAAAGGAGGATAACCATATGTGTTGGGCAGCCGCAGTACCTATCGCTAGATCTGGCGCTCAGGCTATCAGTGATCAGAACGCTCAGGCCAAAATGATTGCCGCTCAGACCGCTGCTGGTCGTCGTCAAGCTATGGAAATCATGAGGCAGACGAACATCCAGAATGCTGACCTATCGTTGCAAGCTCGAAGTAAACTTGAGGAAGCGTCCGCCGGGTTGACACCACAGAACATGCAGAAGGTCCAAGCTATTGGGTCTATCCGAGCg                                                                                                                                                                                                                                                               <  1:460166/301‑1 (MQ=255)`  
                                            `aTGGTCAATTTCAGTTATTTACAATCACGAAAGGAGGATAACCATATGTGTTTGGCAGCCGCAATACCTATCGCTATATCTGGCGCTCAGGCTATCAGTGATCAGAACGCTCAGGCCAAAATGATTGCCGCTCAGACCGATGCTGGTCGTCGTCAAGCTATGGAAATCATGAGGCAGACGAACATCCAGAATGCTGACCTATCGTTGCAAGCTCGAAGTAAACTTGAGGAAGCGTCCGCCGAGTTGACCCCACAGAACATGCAGAAGGTACAAGCTATTGGGTCTATCCGAGCGGCTATCg                                                                                                                                                                                                                                                        >  1:337239/1‑301 (MQ=255)`  
                                              `ggTCAATTTCAGTTATTTACAATCACGAAAGGAGGATAACCATATGTGTTGGGCAGCCGCAATACCTATCGCTATATCTGGCGCTCAGGCTATCAGTGATCAGAACGCTCAGGCCAAAATGATTGCCGCTCAGACCGCTGCTGGTCGTCGTCAAGCTATGGAAATCATGAGGCAGACGAACATCCAGAATGCTGACCTATCGTTGCAAGCTCGAAGTAAACTTGAGGAAGCGTCCGCCGAGTTGACCCCACAGAACATGCAGAAGGTCCAAGCTATTGGGTCTATCCGAGCGGCTATCGga                                                                                                                                                                                                                                                      >  1:231797/1‑301 (MQ=255)`  
                                               `gTCAATTTCAGTTATTTACAATCACGAAAGGAGGATAACCATATGTGTTTGGCAGCCGCAATACCTATCGCTATATCTGGCGCTCAGGCTATCAGTGATCAGAACGCTCAGGCCAAAATGATTGCCGCTCAGACCGCTGCTGGTCGTCGTCAAGCTATGGAAATCATGAGGCAGACGAACATCCAGAATGCTGACCTATCGTTGCAAGCTCGAAGTAAACTTGAGGAAGCGTCAGCCGAGTTGACCCCACAGAACATGCAGAAGGTCCAAGCTATTGGGTCTATCCGAGCGGCTATCGgag                                                                                                                                                                                                                                                     >  1:716087/1‑301 (MQ=255)`  
                                               `gTCAAATTCAGTTAATTACAATCACGAAAGGATGATAACCATATGTGTTGGGCAGCCGCAATACCTATCGCTATATCTGGCGCTCAGGCTATCAGTGGTCAGAACGCTCAGGCCAAAATGAGTGCCGCTCAGACCGCTGCTGGTCGTCGTCAAGCTATGGAAATCATGAGGCAGACGAACATCCAGAATGCTGACCTATCGTTGCAAGCTCGAAGTAAACTTGAGGAAGCGTCCGCCGAGTTTACCCCACAGAACATGCAGAAGGTCCAAGCTATTGGGTCTATCCGAGCGGCTATCGgag                                                                                                                                                                                                                                                     <  1:517721/301‑1 (MQ=255)`  
                                               `acgtgtataagagacagggaggataaccatatgtgttgggcagccgcaatacctatcgctatatctggCGCTATATCTGGCGCTCAGGCTATCAGTAGTCAGAACGCTCAGGCCAAAATGATTGCCGCTCAGACCGCTGCTGGTCGTCGTCAAGCTATGGAAATCATGAGGCAGACGAACATCCAGAATGCTGACCTATCGTTGCAAGCTCGAAGTAAACTTGAGGAAGCGTCCGCCGAGTTGACCTCACAGAACATGCAGAAGGTCCAAGCTATTGGGTCTATCCGAGCGGCTATCagag                                                                                                                                                                                                                                                     <  1:188655‑M2/233‑1 (MQ=255)`  
                                                 `tttacaatcacgaaaggaggataaccatatgtgttgggcatccgcaatacctatcgctatatctggCGCTATATCTGGCGCTCAGGCTATTAGTGGTCAGAACGCTCAGGCCAAAATGATTGCCGCTCAGACCGCTGCTGGTCGTCGTCAAGCTATGGAAATCATGAGGCAGACGAACATCCAGAATGCTGACCTATCGTTGCAAGCTCGAAGTAAACTTGAGGAAGCGTCCGCCGAGTTGACCCCACAGAACATGCAGAAGGTCCAAGCTATTGGGTCTATCCGAGCGGCTATCGgagag                                                                                                                                                                                                                                                   <  1:500575‑M2/235‑1 (MQ=255)`  
                                                 `taaTTTCAGTTATTTACAATCACGAAAGGAGGATAACCATATGTGTTGGGCAGCCGCAATACCTATCGCTATATCTGGCGCTCAGGCTATCAGTGATCAGAACGCTCAGGCCAAAATGATTGCCGCTCAGACCGCTGCTGGTCGTCGTCAAGCTATGGAAATCATGAGGCAGACGAACATCCAGAATGCTGACCTATCGTTGCAAGCTCGAAGTAAACTTGAGGAAGCGTCCGCCGAGTTGACCCCACAGAACATGCAGAAGGTCCAAGCTATTGGGTCTATCCGAGCGGCTATCGgagag                                                                                                                                                                                                                                                   <  1:447081/300‑1 (MQ=255)`  
                                                 `cgtgggctcggagatatgtataagagacagggaTAACCATATGTGTTGGGCAGCCGCAGTACCTATCGCTATATCTGGCGCTCAGGCTATCAGTGATCAGAACGCTCAGGCCAAAATGATTGCCGCTCAGACCGCTGCTGGTCGTCGTCAAGCTATGGAAATCATGAGGCAGACGAACATCCAGAATGCTGACCTATCGTTGCAAGCTCGAAGTAAACTTGAGGAAGCGTCCGCCGAGTTGACCCCACAGAACATGCAGAAGGTCCAAGCTATTGGGTCTATCCGAGCGGCTATCGgagag                                                                                                                                                                                                                                                   <  1:512912/271‑1 (MQ=255)`  
                                                   `ggctcggagatgtgtataagagacaggaggATAACCATATGTGTTGGGCAGCCGCAATACCTATCGCTATATCTGGCGCTCAGGCTATCAGTGATAAGAACGCTCAGGCCAAAATGATTGCCGCTCAGACTGCTGCTGGTCGTCGTCAAGCTATGGAAATCATGAGGCAGACGAACATCCAGAATGCTGACCTATCGTTGCAAGCTCGAAGTAAACTTGAGGAAGCGTCAGCCGAGTTGACCCCACAGAACATGCAGAAGGTCCAAGCTATTGGGTCTATCCGAGCGGCTATCGGagagag                                                                                                                                                                                                                                                 <  1:402729/277‑1 (MQ=255)`  
                                                   `aTTTCAGTTATTTACAATCACGAAAGGAGGATAACCATATGTGTTGGGCAGCCGCAATACCTATCGCTATATCTGGCGCTCAGGCTATCAGTGATCAGAACGCTCAGGCCAAAATGATTGCCGCTCAGACCGCTGCTGGTCGTCGTCAAGCTATGGATATCATGAGGCAGACGAAAATCCAGAATGCTGACCTATCGTTGCAAGCTCGAAGTAAACTTGAGGAAGCGTCCGCCGAGTTGACCCCACAGAACATGCAGAAGGTCCAAGCTATTGGGTCTATCCGAGCGGCTATCGGagagag                                                                                                                                                                                                                                                 >  1:68982/1‑301 (MQ=255)`  
                                                    `tttCAGTTATTTACAATCACGAAAGGAGGATAACCATATGTGTTGGGCAGCCGCAATACCTATCGCTATATCTGGCGCTCAGGCTATCAGTGATCAGAACGCTCAGGCCAAAATGATTGCCGCTCAGACCGCTGCTGGTAGTCGTCAAGCTATGGAAATCATGAGGCAGACGAACATCCAGAATGCTGACCTATCGTTGCAAGCTCGAAGTAAACTTGAGGAAGCATCCGCCGAGTTGACCCCACAGAACATGCAGAAGGTCCAAGCTATTGGGTCTATACAAGCGGCTATCGgagaggga                                                                                                                                                                                                                                                >  1:603204/1‑298 (MQ=255)`  
                                                     `agatgtgtataagagacagaccatatgtgttgggcagccgcaatacctatcgctatatctggCGCTATATCTGGCGCTCAGGCTATCAGTGGTCAGAACGCTCAGGCCAAAATGATTGCCGCTCAGACCGCTGCTGGTCGTCGTCAAGCTATGGAAATCATGAGGCAGACGAACATCCAGAATGCTGACCTATCGTTGCAAGCTCGAAGTAAACTTGAGGAAGCGTCCGCCGAGTTGACCTCACAGAACATGCAGAAGGTCCAAGCTATTGGGTCTATCCGAGCGGCTATCGGAGAGAGTa                                                                                                                                                                                                                                               <  1:427269‑M2/239‑1 (MQ=255)`  
                                                        `ggggcgcggagatgtgtataagagacagaCCATATGTGTTGGGCAGCCGCAATACCTATCGCTATATCTGGCGCTCAGGCTATCAGTGATCAGAACGCTCAGGCCAAAATGATTGCCGCTCAGACCGCTGCTGGTCGTCGTCAAGCTATGGAAATCATGAGGCAGACGAACATCCAGAATGCTGACCTATCGTTGCAAGCTCGAAGTAAACTTGAGGAAGCGTCCGCCGAGTTGACCCCACAGAACATGCAGAAGGTCCAAGCTATTGGGTCTATCCGAGCGGCTATCGGAGAGAGTATGc                                                                                                                                                                                                                                            <  1:284969/273‑1 (MQ=255)`  
                                                         `taagagacagaTCATGAAAGGAGGATAACCATATGTGTTGGGCAGCCGCAATACCTATCGCTATATCTGGCGCTCAGGCTATCAGTGATCAGAACGCTCAGGCCAAAATGATTGCCGCTCAGACCGTTGCTGGTCGTCGTCAAGCTATGGAAATCATGAGGCAGACGAACATCCAGAATGCTGACCTATCGTTGCAAGCTCGAAGTAAACTTGAGGAAGCGTCCGCCGAGTTGACCCCACAGAACATGCAGAAGGTCCAAGCTATTGGGTATATCCGAGCGGCTATCGGAGAGAGTATGCt                                                                                                                                                                                                                                           <  1:572178/291‑1 (MQ=255)`  
                                                         `gTTATTTACAATCACGAAAGGAGGATAACCATATGTGTTGGGCAGCCGCAATACCTATCGCTATATCTGGCGCTCAGGCTATCAGTGATCAGAACGCTCAGGCCAAAATGATTGTCGCTCAGACCGCTGCTGGTCGTCGTCAAGCTATGGAAATCATGAGGCAGACGAACATCCAGAATGCTGACCTATCGTTGCAAGCTCGAAGTAAACTTGAGGAAGCGTCCGCCGAGTTGACCCCACAGAACATGCAGAAGGTCCAAGCTATTGGGTCTATCCGAGCGGCTATCGGAGAGAGTATGCt                                                                                                                                                                                                                                           <  1:588895/301‑1 (MQ=255)`  
                                                                     `cACGAAATGAGGATAACCATATGTGTTGGGCAGCCGCAATACCTATCGCTATATCTGGCGCTCAGGCTATCAGTGGTCAGAACGCTCAGGCCAAAATGATTGCCGCTCAGACCGCTGCTGGTCGTCGTCAAGCTATGGAAATAATGAGGCAGACGAACATCCAGAATGCTGACCTATCGTTGCAAGCTCGAAGTAAACTTGAGGAAGCGTCCGCCGAGTTGACCTAACAGAACATGCAGAAGGTCCAAGCTATTGGGTCTATCCGAGCGGCTATCGgagagctgtctcttatacacatctc                                                                                                                                                                                                                               >  1:259076/1‑281 (MQ=255)`  
                                                                     `cACGAAAGGAGGATAACCATATGTGTTGGGCAGCCGCAATACCTATCGCTATATCTGGCGCTCAGGCTATCAGTGATCAGAACGCTAAGGCCAAAATGATTGCCGCTCAGACCGCTGCTGGTCGTCGTCAAGCTATGGAAATCATGAGGCAGACGAACATCCAGAATGCTGACCTATCGTTGCAAGCTCGAAGTAAACTTGAGGAAGCGTCCGCCGAGTTGACCCCACAGAACATGCAGAAGGTCCAAGCTATTGGGTCTATCCGAGCGGCTATCGGAGAGAGTATGCCTgtctcttatac                                                                                                                                                                                                                               >  1:495046/1‑291 (MQ=255)`  
                                                                               `gtgttgggcagccgcaatacctatcgctatatctggCGCTATATCTGGCGCTCAGGCTATCAGTGGTCAGAACGCTCAGGCCAAAATGATTGCCGCTCAGACCGCTGCTGGTCGTCGTCAAGCTATGGAAATCATGAGGCAGACGAACATCCAGAATGCTGACCTATCGTTGCAAGCTCGAAGTAAACTTGAGGAAGCGTCCGCCGAGTTGACCCCACAGAACATGCAGAAGGTCCAAGCTATTGGGTCTATCCGAGCGGCTATCGGAGAGAGTATGCTTGAAGGTTCCTCAATGGACCGc                                                                                                                                                                                                                     >  1:639752‑M2/37‑301 (MQ=255)`  
                                                                                 `gagatgtgtataagagaccggCCGCAATACCTATCGCTATATCTGGCGCTCAGGCTATCAGTGATCAGAACGCTCAGGCCAAAATGATTGCCGCTCAGACCGCTGCTGGTCGTCGTCAAGCTATGGAAATCATGAGGCAGACGAACATCCAGAATGCTGACCTATCGTTGCAAGCTCGAAGTAAACTTGAGGAAGCCTCCGCCGAGTTGACCCCACAGAACATGCAGAAGGTCCAAGCTTTTGGGTCTATCCGAGCGGCTATCGGAGAGAGTATGCTTGAAGGTTCCTCAATGGACCGCAt                                                                                                                                                                                                                   <  1:563046/281‑1 (MQ=255)`  
                                                                                   `aaCCATATGTGTTGGGCAGCCGCAATACCTATCGCTATATCTGGCGCTCAGGCTATCAGTGATCAGAACGCTCAGGCCAAAATGATTGCCGCTCAGACCGCTGCTGGTCGTCGTCAAGCTATGGAAATCATTAGGCAGACGAACATCCAGAATGCTGACCTATCGTTGCAAGCTCGAAGTAAACTTGAGGAAGCGTCCGCCGAGTTGACCCCACAGAACATGCAGAAGGTCCAAGCTATTGGGTCTATCCGAGCGGCTATCGGAGAGAGTATGCTTGAAGGTTCCTCaactgtctcttata                                                                                                                                                                                                                 >  1:624986/1‑289 (MQ=255)`  
                                                                                               `atacctatcgctatatctggCGCTATATCTGGCGCTCAGGCTATCAGTGGTCAGAATGCTCAGGCCAAAATGATTGCCGCTCAGACCGCTGCTGGTCGTCGTCAAGCTATGGAAATCATGAGGCAGACGAACATCCAGAATGCTGACCTATCGTTGCAAGCTCGAAGTAAACTTGAGGAAGCGCCCGCCGAGTTGACCACACAGAACATGCAGAAGGTCCAAGCTATTGGGTGTATCCGAGCGGCTATCGGATAGAGTATGCTTGAAGGTTCATCAATGGAGCGCATTAAGCGAGTCACAg                                                                                                                                                                                                     >  1:246‑M2/21‑301 (MQ=255)`  
                                                                                                     `gCCGCAATACCTATCGCTATATCTGGCGCTCAGGCTATCAGTGGTCAGAACGCTCAGGCCAAAATGATTGCCGCTAAGACCGCTGCTGGTCGTCGTCAAGCTATGGAAATCATGAGGCAGACGAACATCCAGAATGCTGACCTATCGTTGCAAGCTCGAAGTAAACTTGAGGAAGCGTCCGCCGAGTTGACCTCACAGAACATGCAGAAGGTACAAGCTATTGGGTCTATCCGAGCGGCTATCGGAGAGAGTATGCTTGAAGGTTCCTCAATGGACCGCATTAAGCGAGGCACAGAAGGAc                                                                                                                                                                                               >  1:439780/1‑301 (MQ=255)`  
                                                                                                     `gCCGCAATACCTATCGCTATATCTGGCGCTCAGGCTATCAGTGATCAGAACGCTCAGGCCAAAATGATTGCCGCTCAGACCGCTGCTGGTCGTCGTCAAGCTATGGAAATCATGATGCAGACGAACATCCAGAATGCTGACCTATCGTTGCAAGCTCGAAGTAAACTTGAGGAAGCGTCCGCCGAGTTGACCCCACAGAACATGCAGAAGGTCCAAGCTATTGGGTCTATCCGAGCGGCTATCGGAGAGAGTATGCTTGAAGGTTCCTCAATGGACCGCATTAAGCGAGTCACAGAAGGAc                                                                                                                                                                                               <  1:194456/301‑1 (MQ=255)`  
                                                                                                     `atcgctatatctggCGCTATATCTGGCGCTCAGGCTATCAGTGGTCAGAACGCTCAGGCCAAAATGATTGCCACTCAGACCGCTGCTGGTCGTCGTCAAGCTATGGAAATCATGAGGCAGACGAACATCCAGAATGCTGACCTATCGTTGCAAGCTCGAAGTAAACTTGAGGAAGCGTCCGCCGAGTTGACCCCACAGAACATGCAGAAGGTCCAAGCTATTGGGTCTATCCGAGCGGCTATCGGAGAGAGTATGCTTGAAGGTTCCTCAATGGACCGCATTAAGCGAGTCACAGAAGGAc                                                                                                                                                                                               <  1:415589‑M2/287‑1 (MQ=255)`  
                                                                                                       `cGCAATACCTATCGCTATATCTGGCGCTCAGGCTATCAGTGATCAGAACGCTCAGTCCAAAATGATTGCCGCTCAGACCGCTGCTGGTCGTCGTCAAGCTATGGAAATCATGAGGCAGACGAACATCCAGAATGCTGACCTATCGTTGCAAGCTCGAAGTAAACTTGAGGAAGCGTCCGCCGAGTTGACCCCACAGAACATGCAGAAGGTCCAAGCTATTGGGTCTATCCGAGCGGCTATCGGAGAGAGTATGCTTGAAGGTTCCTCAATGGACCGCATTAAGCGAGTCACAGAAGGACAg                                                                                                                                                                                             >  1:279620/1‑301 (MQ=255)`  
                                                                                                        `gCAATACCTATCGCTATATCTGGCGCTCAGGCTATCAGTGATCAGAACGCTCAGGCCAAAATGATTGCCGCTCAGACCGCTGCTGGTCGTCGTCAAGCTATGGAAATCATGAGGCAGACGAACATCCAGAATGCTGACCTATCGTTGCAAGCTCGAAGTAAACTTGAGGAAGCGTCCGCCGAGTTGACCCCACAGAACATGCAGAAGGTCCAAGCTATTGGGTCTATCCGAGCGGCTATCGGAGAGAGTATGCTTGAAGGTTCCTCAATGGACCGCATTAAGCGAGTCACAGAAGGACAGt                                                                                                                                                                                            >  1:817891/1‑301 (MQ=255)`  
                                                                                                             `atgtgtataagagacaggCGCTCAGGCTATCAGTGGTCAGAACGCTCAGGCCAAAATGATTGCCGCTCAGACCGCTGCTGGTCGTCGTCAAGCTATGGAAATCATGAGGCAGACGAACATCCAGAATGCTGACCTATCGTTGCAAGCTCGAAGTAAACTTGAGGAAGCGTCCGCCGAGTTGACCACACAGAACATGCAGAAGGTCCAAGCTATTGGGTCTATCCGAGCGGCTATCGGAGAGAGTATGCTTGAAGGTTCCTCAATGGACCGCATTAAGCGAGTCACAGAAGGACAGTTCAtt                                                                                                                                                                                       <  1:791680/285‑1 (MQ=255)`  
                                                                                                                 `aTCGCTATATCTGGCGCTCAGGCTATCAGTGATCAGAACGCTCAGGCCAAAATGATTGCCGCTCAGACCGCTGCTGGTCGTCGTCAAGCTATGGAAATCATGAGGCAGACGAACATCCAGAATGCTGAACTATCGTTGCAAGCTCGAAGTAAACTTGAGGAAGCGTCTGCCGAGTTGACCCCACAGAACATGCAGAAGGTCCAAGCTATTGGGTCTATCCGAGCGGCTATCGGAGAGAGTATGCTTGAAGGTTCCTCAATGGACCGCATTAAGCGAGTCACAGAAGGACAGTTCATTCggg                                                                                                                                                                                   >  1:175195/1‑301 (MQ=255)`  
                                                                                                                  `gCGCTATATCTGGCGCTCAGGCTATCAGTGGTCAGAATGCTCAGGCCAAAATGATTGCCGCTCAGACCGCTGCTGGTCGTCGTCAAGCTATGGAAATCATGAGGCAGACGAACATCCAGAATGCTGACCTATCGTTGCAAGCTCGAAGTAAACTTGAGGAAGCGTCCGCCGAGTTGACCTCACAGAACATGCAGAAGGTCCAAGCTATTGGGTCTATCCGAGCGGCTATCGGAGAGAGTATGCTTGAAGGTTCCTCTATGGACCGCATTAAGCGAGTCACAGAAGGCCAGTTCATTCTGGa                                                                                                                                                                                  >  1:700595‑M2/2‑301 (MQ=255)`  
                                                                                                                    `gCTATATCTGGCGCTCAGGCTATCAGTGATCAGAACGCTCAGGCCAAAATGATTGCCGCTCAGACCGCTGCTGGTCGTCGTCAAGCTATGTAAATCATGAGGCAGACGAACATCCAGAATGCTGACCTATCGTTGCAAGCTCTAAGTAAACTTGAGGAAGCGTCCGCCGAGTTGACCCCACAGAACATGCAGAAGGTCCAAGCTATTGGGTCTATCCGAGCGGCTATCGGAGAGAGTATGCTTGAAGGTTCCTCAATGGACCGCATTAAGCGAGTCACAGAAGGACAGTTCATTCgggctg                                                                                                                                                                                >  1:620962/1‑298 (MQ=255)`  
                                                                                                                     `cTATATCTGGCGCTCAGGCTATCAGTGATCAGAACGCTCAGGCCAAAATGATTGCCGCTCAGACCGCTGCTGGTCGTCGTCAAGCTATGGAAATCATGAGGCAGACGAACATCCAGAATGCTGACCTATCGTTGCAAGCTCGAAGTAAACTTGAGGAAGCGTCCGCCGAGTTGACCCCACAGAACATGCAGAAGGTCCAAGCTATTGGGTCTATCAGAGCGGCTATCGGAGAGAGTATGCTTGAAGGTTCCTAAATGGACCGCATTAAGCGAGTCACAGAAGGACAGTTCATTCGGGAAGc                                                                                                                                                                               >  1:454443/1‑301 (MQ=255)`  
                                                                                                                     `cTATATCTGGCGATCAGGCTATCAGTGATCAGAACGCTCAGGCCAAAATGATTGCCGCTCAGACCGCTGCTGGTCGTCGTCAAGCTATGGAAATCATGAGGCAGACGAACATCCAGAATGCTGACCTATCATTGCAAGCTCGAAGTAAACTTGAGGAAGCGTCCGCCGAGTTGACCCCACAGAACATGCAGAAGGTCCAAGCTATTGGGTCTATCCGAGCGGCTATCGGAGAGAGTATGCTTGAAGGTTCCTCAATGGACCGCATTAAGCGAGTCACAGAAGGACAGTTCATTCGGGAAGc                                                                                                                                                                               >  1:8953/1‑301 (MQ=255)`  
                                                                                                                              `gcgcTCATGCTATCAGTGATCAGAACGCTCAGGCCAAAATGATTGCCGCTCAGACCGCTGCTGGTCGTCGTCAAGCTATGGAAATCATGAGGCAGACGAACATCCAGAATGCTGACCTATCGTTGCAAGCTCGAAGTAAACTTGAGGAAGCGTCCGCCGAGTTGACCCCACAGAACATGCAGAAGGTCCAAGCTATTGGGTCTATCCGAGCGGCTATCGGAGAGAGTATGCTTGAAGGTTCCTCAATGGACCGCATTAAGCGAGTCACAGAAGGACAGTTCATTCGGGAAGCCAATATggc                                                                                                                                                                      >  1:778172/1‑300 (MQ=255)`  
                                                                                                                              `gcgcTCAGGCTATCAGTGGTCAGAACGCTCAGGCCAAAATGATTGCCGCTCAGACCGATGCTGGTCGTCGTCAAGCTATGGAAATCATGAGGCAGACGAACATCCAGAATGCTGACCTATCGTTGCAAGCTCGAAGTAAACTTGAGGAAGCGTCCGCAGAGTTGACCACACAGAACATGCAGAAGGTCCAAGCTATTGGGTCTATCCGAGCGGCTATCGGAGAGAGTATGCTTGAAGGTTCCTCAATGGACCGCATTAAGCGAGTCACAGAAGGACAGTTCATTCGGGAAGCCAATATGGt                                                                                                                                                                      <  1:164726/301‑1 (MQ=255)`  
                                                                                                                                 `tgggctcggagatgtgtataagagacagggCCAAAATGATTGCCGCTCAGACCGCTGCTGGTCGTCGTCAAGCTATGGAAATCATGAGGCAGACGAACATCCAGAATGCTGACCTATCGTTGCAAGCTCGAAGTAAACTTGAGGAAGCGTCCGCCGAGTTGACCCCACAGAACATGCAGAAGGTCCAAGCTATTGGGTCTATCCGAGCGGCTATCGGAGAGAGTATGCTTGAAGGTTCCTCAATGGACCGCATTAAGCGAGTCACAGAAGGACAGTTCATTCGGGAAGCCAATATGGTAAc                                                                                                                                                                   <  1:550411/273‑1 (MQ=255)`  
                                                                                                                                        `tATCAGTGATCAGAACGCTCAGGCCAAAATGATTGCCGCTCAGACCGCTGCTGGTCGTCGTCAAGCTATGGAAATCATGAGGCAGACGAACATCCAGAATGCTGACCTATCGTTGCAAGCTCGAAGTAAACTTGAGGAAGCGTCCGCCGAGTTGACCACACAGAACATGCAGAAGGTCCAAGCTATTGGGTCTATCCGAGCGGCTATCGGAGAGAGTATGCTTGAAGGTTCCTCAATGGACCGCATTAAGCGAGTCACAGAAGGACAGTTCATTCGGGAAGCCAATATGGTAACTGAGAAc                                                                                                                                                            <  1:756438/301‑1 (MQ=255)`  
                                                                                                                                                `gtataagagacagggCCAAAATGATTGCCGCTCAGACCGCTGCTGGTCGTCGTCAAGCTATGGAAATCATGAGGCAGACGAACATCCAGAATGCTGACCTATCGTTGCAAGCTCGAAGTAAACTTGAGGAAGCGTCCGCCGAGTTGACCCCACAGAACATGCAGAAGGTCCAAGCTATTGGGTCTATCCGAGCGGCTATCGGAGAGAGTATGCTTGAAGGTTCCTCAATGGACCGCATTAAGCGAGTCACAGAAGGACAGTTCATTCGGGAAGCCAATATGGTAACTGAGAACTATCGcca                                                                                                                                                    <  1:102879/288‑2 (MQ=255)`  
                                                                                                                                                `atCAGAACGCTCAGGCCAAAATGATTGCCGCTCAGACCGCTGCTGGTCGTCGTCAAGCTATGGAAATCATGAGGCAGACGAACATCCAGAATGCTGACCTATCGTTGCAAGCTCGAAGTAAACTTGAGGAAGCGTCCGCCGAGTTGACCCCACAGAACATGCAGAAGGTCCAAGCTATTGGGTCTATCCGAGCGGCTATCGTAGAGAGTATGCTTGAAGGTTCCTCAATGGACCGCATTAAGCGAGTCACAGAAGGACAGTTCATTCGGGAAGCCAATATGGTAACTGAGAACTATCGCCg                                                                                                                                                    <  1:540133/300‑1 (MQ=255)`  
                                                                                                                                                 `tCAGAACGCTCAGGCCAAAATGATTGCCGCTCAGACCGCTGCTGGTCGTCGTCAAGCTATGGAAATCATGAGGCAGACGAACATCCAGAATGCTGACCTATCGTTGCAAGCTCGAAGTAAACTTGAGGAAGCGTCCGCCGAGTTGACCCCACAGAACATGCAGAGGGTCCAAGCTATTGGGTCTATCCGAGCGGCTATCGGAGAGAGTATGCTTGAAGGTTCCTCAATGGACCGCATTAAGCGAGTCACAGAAGGACAGTTCATTCGGGAAGCCAATATGGTAACTGAGAACTATCGCCGt                                                                                                                                                   >  1:185681/1‑301 (MQ=255)`  
                                                                                                                                                              `gctcggagatgtgtataagaGACAGCTGCTGGTCGTCGTCAAGCTATGGAAATCATGAGGCAGACGAACATCCAGAATGCTGACCTATCGTTGCAAGCTCGAAGTAAACTTGAGGAAGAGTCCGCCGAGTTGACCCCACAGAACATGCAGAAGGTCCAAGCTATTGGGTCTATCCGAGCGGCTATCGGAGAGAGTATGCTTGAAGGTTCCTCAATGGACCGCATTAAGCGAGTCACAGAAGGACAGTTCATTCGGGAAGCCAATATGGTAAATGAGAACTATCGCCGTGACTACCAAGCaa                                                                                                                                      <  1:299779/282‑1 (MQ=255)`  
                                                                                                                                                              `gacAAAATGATTGCCGCTCAGACCGCTGCTGGTCGTCGTCAAGCTATGGAAATCATGAGGCAGACGAACATCCAGAATGCTGACCTATCGTTGCAAGCTCGAAGTAAACTTGAGGAAGCGTCCGCCGAGTTGACCCCACAGAACATGCAGAAGGTCCAAGCTATTGGGTCTATCCGAGCGGCTATCGGAGAGAGTATGCTTGAAGGTTCCTCAATGGACCGCATTAAGCGAGTCACAGAAGGACAGTTAATTCGGGAAGCCAATATGGTAACTGAGAACTATCGCCGTGACTACCAAGCaa                                                                                                                                      <  1:168585/299‑1 (MQ=255)`  
                                                                                                                                                                     `ggATTGCCGCTCAGACCGCTGCTGGTCGTCGTCAAGCTATGGAAATCATGAGGCAGACGAACATCCAGAATGCTGACCTATCGTTGCAAGCTCGAAGTAAACTTGAGGAAGCGTCCGCCGAGTTGACCTCACAGAACATGCAGAAGGTCCAAGCTATTGGGTCTATCCGAGCGGCTATCGGAGAGAGTATGCTTGAAGGTTCCTCAATGGACCGCATTAAGCGAGTCACAGAAGGACAGTTCATTCGGGAAGCCAATATGGTAACTGAGAACTATCGCCGTGACTACCAAGCAATATTCGc                                                                                                                               <  1:316760/300‑1 (MQ=255)`  
                                                                                                                                                                        `ttGCCGCTCAGACCGCTGCTGGTCGTCGTCAAGCTATGGAAATCATGAGGCAGACGAACATCCAGAATGCTGACCTATCGTTGCAAGCTCGAAGTAAACTTGAGGAAGCGTCCGCCGAGTTGACCCCACAGAACATGCAGAAGGTCCAAGCTATTGGGTCTATCCGAGCGGCTATCGGAGAGAGTATGCTTGAAGGTTCCTCAATGGACCGCATTAAGCGAGTCACAGAAGGACAGTTCATTCGGAAAGCCAATATGGTAACTGAGAACTATCGCCGTGACTACCAAGCAATCTTCGcaca                                                                                                                            >  1:782935/1‑301 (MQ=255)`  
                                                                                                                                                                             `ccTCAGACCGCTGCTGGTCGTCGTCAAGCTATGGAAATCATGAGGCAGACGAACATCCAGAATGCTGACCTATCGTTGCAAGCTCGAAGTAAACTTGAGGAAGCGTCCGCCGAGTTGACCCCACAGAACATGCAGAAGGTCCAAGCTATTGGGTCTATCCGAGCGGCTATCGGAGAGAGTATGCTTGAAGGTTCCTCAATGGACCGCATTAAGCGAGTCACAGAAGGACAGTTCATTCGGGAAGCCAATATGGTAACTGAGAACTATCGCCGTGACTACCAAGCAATCTTAGCACAGCAAc                                                                                                                       <  1:373615/300‑1 (MQ=255)`  
                                                                                                                                                                                   `cccGCTGCTGGTCGTCGTCAAGCTATGGAAATCATGCTGCAGACGAACATCCAGAATGCTGACCTATCGTTTCAAGCTCGAAGTAACCTTGAGGAAGCGTCCGCCGAGTTGACCCCACAGAACATGCAGCAGGTCCAAGCTATTGGGTCTATCCGAGCGGCTATCGGAGAGAGTATGCTTGAAGGTTCCTCAATGTACCGCATTAAGCGAGTCACAGAAGGACAGTTCTTTCGGGAAGCCAATATGGTAACTGAGAACTATCGCCGTGACTACCAAGCAATCTTCGCACAGCAACTtggtg                                                                                                                 <  1:759203/300‑1 (MQ=255)`  
                                                                                                                                                                                    `cctctgctgGGCGTCGTCAAGCTATGGAAATCATGAGACAGACGAACATCCAGAATGCTGACCTATCGTTGCAAGCTCGAAGTAAACTTGAGGAAGCGTCCGCCGAGTTTACCCCACAGAACATGCAGAAGGTCCAAGCTATTGGGTCTATCCGAGCGGCTATCGTAGAGAGTATGCTTGAAGGATCCTCAATGGACCGCATTAAGCGAGTCACAGAAGGACAGTTCATTCGGGAAGCCAATATGGTAACTTAGAACTATCGCCGTGACTACCAAGCAATCGTCGCACAGCAACTtggtgg                                                                                                                <  1:362942/298‑1 (MQ=255)`  
                                                                                                                                                                                    `agatgtgtataagagacagaGCTATGGAAATCATGAGGCAGACGAACATCCAGAATGCTGACCTATCGTTGCAAGCTCGAAGTAAACTTGAGGAAGCGTCCGCCGAGTTGACCCAACAGAACATGCAGAAGGTCCAAGCTATTGGGTCTATCCGAGCGGCTATCGGAGAGAGTATGCTTGAAGGTTCCTCAATGGACCGCATTAAGCGAGTCACAGAAGGACAGTTCATTCGGGAAGCCAATATGGTAACTGAGAACTATCGCCGTGACTACCAAGCAATCTTCGCACAGCAACTtggtgg                                                                                                                <  1:532470/282‑1 (MQ=255)`  
                                                                                                                                                                                       `ctgctgGTCGTCGTCAAGCTATGGAAATCATGAGGCAGACGAACATCCAGAATGCTGACCTATCGTTGCAAGCTCGAAGTAAACTTGAGGAAGCATCCGCCGAGTTGACCCCACAGAACATGCAGAAGGTCCAAGCTATTGGGTCTATCCGAGCGGCTATCGGAGAGAGTATGCTTGAAGGTTCCTAAATGGACAGCATTAAGCGAGTCACAGAAGGACAGTTCATTCGGGAAGCCAATATGGTAACTGAGAACTATCGCAGTGACTACCAAGCAATCTTCGCACAGCAACTTGGTAGTAc                                                                                                             <  1:555757/301‑1 (MQ=255)`  
                                                                                                                                                                                         `gctgGTCGTCGTCAAGCTATGGAAATCATGAGGCAGACGAACATCCAGAATGCTGACCTATCGTTGCAAGCTCGAAGTAAACTTGAGGAAGCGTCCGCCGAGTTGACCCCACAGAACATGCAGAAGGTCCAAGCTATTGGGTCTATCCGAGCGGCTATCGGAGAGAGTATGCTTGAAGGTTCCTCAATGGACCGCATTAAGCGAGTCACAGAAGGACAGTTCATTCGGGAAGCCAATATGGTAACTGAGAACTATCGCCGTGACTACCAAGCAATCTTCGCACATCAACTTGGTGGTACTc                                                                                                           <  1:323917/301‑1 (MQ=255)`  
                                                                                                                                                                                         `gctgGTCGTCGTCAAGCTATGGAAATCATGAGGCAGACGAACATCCAGAATGCTGACCTATCGTTGCAAGCTCAAAGTAAACTTGAGGAAGCGTCCGCCGAGTTGACCCCACAGAACATGCAGAAGGTCCAAGCTATTGGGTCTATCCGAGCGGCTATCGGAGAGAGTATGCTTGAAGGTTCCTCAATGGACCGCATTAAGCGAGTCACAGAAGGACAGTTCATTCGGGAAGCCAATATGGTAACTGAGAACTATCGCCGTGACTACCAAGCAATCTTCGCACAGCAACTTGGTGGTACTc                                                                                                           <  1:114710/301‑1 (MQ=255)`  
                                                                                                                                                                                              `tcgtcgtcAAGCTATGGAAATCATGAGGCAGACGAACATCCAGAATGCTGACCTATCGGTGCAAGCTCGAAGTAAACTTGAGGAAGCGTCCGCCGAGTTGACCCAACAGAACATGCAGAAGGTCCAAGCTATTGGGTCTATCCGAGCGGCTATCGGAGAGAGTATGCTTGAAGGTTCCTCAATGGACCGCATTAAGCGAGTCACAGAAGGACAGTTCATTCGGGAAGCCAATATGGTAACTGAGAACTATCGCCGTGACTACCAAGCAATCTTAGCACAGCAACTTGGTGGTACTCAAAGt                                                                                                      <  1:6179/301‑1 (MQ=255)`  
                                                                                                                                                                                              `tcgtcgtcAAGCTAGGGAAAGCAGGAGGCAGACGAACATCCAGAATGCTGACCTATCGTTGCAAGCTCGAAGTAAACTTGAGGAAGCGTCCGCCGAGTTGACCCCACAGAACATGCAGAAGGTCCAAGCTATTGGGTCTATCCGAGCGGCTATCGGAGAGAGTATGCTTGAAGGTTCCTCAATGGACCGAATTAAGCGAGTCACAGAAGGACAGTTCATTCGGGAAGCCAATATGGTAACTGAGAACTATCGCCGTGACTACCAAGCAATCTTCGCACAGCAACTTGGTGGTACTCAAAGt                                                                                                      <  1:205696/301‑1 (MQ=255)`  
                                                                                                                                                                                                           `aTGGAAATCATTAGGCAGACGAACATCCAGAATGCTGACCTATCGTTGCAAGCTCGAAGTAAACTTGAGGAAGCGTCCGCCGAGTTGACCCCACAGAACATGCAGAAGGTCCAAGCTATTGGGTCTATCCGAGCGGCTATCGGAGAGAGTATGCTTGAAGGTTCCTCAATGGACCGCATTAAGCGAGTCACAGAAGGACAGTTCATTCGGGAAGCCAATATGGTAACTGAGAACTATCGCCGTGACTACCAAGCAATCTTCGCACAGCAACTTGGTGGTACTCAAAgctgtctcttataca                                                                                         >  1:642682/1‑287 (MQ=255)`  
                                                                                                                                                                                                           `aTGGAAATCATGAGGCAGACGAACATCCAGAATGCTGACCTATCGTTGCAAGCTCGAAGTAAACTTGAGGAAGCGTCCGCAGAGTTGACCCCACAGAACATGCAGAAGGTCCAAGCTATTGGGTCTATCCGAGCGGCTATCGGAGAGAGTATGCTTGAAGGTTCCTCAATGGACCGCATTAAGCGAGTCACAGAAGGACAGTTCATTCGGGAAGCCAATATGGTAACTGAGAACTATCGCCGTGACTACCAAGCAATCTTCGCACAGCAACTTGGTGGTACTCAAAGTGCTGCAAGTCAGa                                                                                         >  1:406923/1‑301 (MQ=255)`  
                                                                                                                                                                                                             `ggAAATCATGGGAACGTCGAACATCCAGAATGCTGACCTATCGTTGACAGCTCAAAGTACACTTGAGGAAGCGTCCGCCGAGTTGACCCCACAGAACATGCAGAAGGTCCAAGCTAGTGGGTCTATCCGAGCAGCTATCGGAGAGAGTATGCTTGAAGGTTCTCCAATGGACCGCATTAAGCGATTCACAGAAGGACAGTTCATACGGGAAGCCAATATGGTAACTGAGAACTATCGCCGTGACTACCAAGCAATCTTCGCACAGCAACTTGGTGGTACTCAAAGTGCTGCAAGTCAGAtt                                                                                       <  1:743403/301‑1 (MQ=255)`  
                                                                                                                                                                                                                              `cGAACTTCCATAATGCTTGCCTCTCGTTGCAAGCTCGAAGTAAACTTGAGGAAGCGTCAGCCGAGTTGACCCCACAGAACATGCAGAAGGGCCAAGCTATTGTGTCTATCCGAGCGGCTATCGGAGAGAGTATGCTTGAAGGTTCCTCAATGGACCGCATTAAGCGAGTCACAGAAGGACAGTTCATTCGGGAAGCCAATATGGTAACTGAGAACTATCGACGTGACTACCAAGCAATCTTCGCACAGCAACTTGGTGGTACTCAAAGTGCTGCAAGTCAGATTGACGAAATCTATAagag                                                                      <  1:43639/301‑1 (MQ=255)`  
                                                                                                                                                                                                                                  `cATCCAGAATGCTGACCTATCGTTGCAAGCTCGAAGTAAACTTGAGGAAGCGTCCGCCGAGTTGACCCCACAGAACATGCAGAAGGTCCAAGCTATTGGGTCTATCCGAGCGGCTATCGGAGAGAGTATGCTTGAAGGTTCCTCAATGGACCGCATTAAGCGAGTCACAGAAGGACAGTTCATTCGGGAAGCCAATATGGTAACTGAGAACTATCGCCGTGACTACCAAGCAATCTTCGCACAGCAACTTGGTGGTACTCAAAGTGCTGCaactgtctcttatacacatctccgagcccac                                                                  >  1:2210/1‑272 (MQ=255)`  
                                                                                                                                                                                                                                   `aTCCAGAATGCTGACCTATCGTTGCAAGCTCGAAGTAAACTTGAGGAAGCGTCCGCCGAGTTGACCCCACAGAACATGCAGAAGGTCCAAGCTATTGGGTCTATCCGAGCGGCTATCGTAGAGAGTATGCTTGAAGGTTCCTCAATGGACCGCATTAAGCGAGTCACAGAAGGACAGTTCATTCGGGAAGCCAATATGGTAACTGAGAACTATCGCCGTGACTACCAAGCAATCTTCGCACAGCAACTTGGTGGTACTCAAAGTGCTGCAAGTCAGATTGACGactgtctcttatacacat                                                                 >  1:134200/1‑284 (MQ=255)`  
                                                                                                                                                                                                                                   `aTCCAGAATGCTGACCTATCGTTGCAAGCTCGAAGTAAACTTGAGGAAGCGTCCGCCGAGTTGACCCCACAGAACATGCAGAAGGTCCAAGCTATTGGGTCTATCCGAGCGGCTATCGGAGAGAGTATGCTTGAAGGTTCCTCAATGGACCGCATTAAGCGAGTCACAGAAGGACAGTTCATTCGGGAAGCCAATATGGTAACTGAGAACTATCGCCGTGACTACCAAGCAATCTTCGCACAGCAACTTGGTGGTACTCAAAGTGCTGCAAGTcctgtctcttatacacatctccgagccc                                                                 >  1:387929/1‑274 (MQ=255)`  
                                                                                                                                                                                                                                              `tGACCTATCGTTGCAAGCTCGAAGTAAACTTGAGGAAGCGTCCGCCGAGTTGACCTCACAGAACATGCAGAAGGTCCAAGCTATTGGGTCTATCCTAGCGGCTATCGGAGAGAGTATGCTTGAAGGTTCCTCAATGGACCGCATTAATCGAGTCACAGAAGGACAGTTCATTCGGGAAGCCAATATGGTAACTGAGAACTATCGCCGTGACTACCAAGCAATCTTCGCACAGCAACTTGGTGGTACTCAAAGTGCTGCAAGTCAGATTGACGAAATCTATAAGAGCGAACAGAAACagaag                                                      <  1:557937/301‑1 (MQ=255)`  
                                                                                                                                                                                                                                               `gACCTATCGTTGCAAGCTCGATGTAAACTTGAGGAAGCGTCCGCCGAGTTGACCCCACAGAACATGCAGAAGGTCCAAGCTATTGGGTCTATCCGAGCGGCTATCGGAGAGAGTATGCTTGAAGGTTCCTCAATGGACCGCATTAAGCGAGTCACAGAAGGACAGTTCATTCGGGAAGCCAATATGGTAACTGAGAACTATCGCCGTGACTACCAAGCAATCTTCGCACAGCAACTTGGTGGTACTCAAAGTGCTGCAAGTCAGATTGACGAAATCTATAAGAGCGAacagaaacagaata                                                     >  1:327530/1‑299 (MQ=255)`  
                                                                                                                                                                                                                                                    `aTCGTTGCAAGCTCGAAGTAAACTTGAGGAAGCGTCCGCCGAGTTGATCCCACAGAACATGCAGAAGGTCCAAGCTATTTGGTCTATCCGAGCGGCTATCGGAGATAGTATGCTTGAAGGTTCCTCAATGGACCGCATTAAGCGAGTCACAGAAGGACAGTTCATTCGGGAAGCCAATATGGTAACTGAGAACTATCGCCGTGACTACCAAGCAATCTTCGCACAGCAACTTGGTGGTACTCAAAGTGCTGCAAGTCAGATTGACGAAATCTATAAGAGAGAACAGAAACAGAAGAGTaac                                                >  1:343831/1‑300 (MQ=255)`  
                                                                                                                                                                                                                                                    `aTCGTTGCAAGCTCGAAGTAAACTTGAGGAAGCGTCCGCCGAGTTGACCCCACAGAACATGCAGAAGGTCCAAGCTATTGGGTCTATCCGAGCGGCTATCGGAGAGAGTATGCTTGAAGGTTACTCAATGGACCGCATTAAGCGAGTCACAGAAGGACAGTTCATTCGGGAAGCCGATATGGTAACTGAGAACTATCGCCGTGACTACCAAGCAATCTTCGCACAGCAACTTGGTGGTACTCAAAGTGCTGCAAGTCAGATTGACGAAATCTATAAGAGCGAACAGAAACAGAAGAGTAAg                                                >  1:146756/1‑301 (MQ=255)`  
                                                                                                                                                                                                                                                    `aTCGTTGCAAGCTCGAAGTAAACTTGAGGAAGCGTCCGCCGAGTTGACCCAACAGAACATGCAGAAGGTCCAAGCTATTGGGTCTATCCGAGCGGCTATCGGAGAGAGTATGCTTGAAGGTTCCTCAATGGACCGCATTAAGCGAGTCACAGAAGGACAGTTCATTCGGGAAGCCAATATGGTAACTGAGAACTATCGCCGTGACTACCAAGCAATCTTCGCACAGCAACTTGGTGGTACTCAAAGTGCTGCAAGTCAGATTGACGAAATCTATAAGAGCGAACAGAAACAGAAGAGTAAg                                                >  1:664932/1‑301 (MQ=255)`  
                                                                                                                                                                                                                                                                       `aaaCTTGAGGAAGCGTCGGCCGAGTTGACCTCACAGAACATGCAGAAGGTCCAAGCTATTGGGTCTATCCGAGCGGCTATCGGAGAGAGTATGCTTGAAGGTTCCTCAATGGACCGCATTAAGCGAGTCACAGAAGGACAGTTCATTCGGGAAGCCAATATGGTAACTGAGAACTATCGCCGTGACTACCAAGCAATCTTCGCACAGCAACTTGGTGGTACTCAAAGTGCTGCAAGTCAGATTGACGAAATCTATAAGAGCGAACAGAAACAGAAGAGTAAGCTACAGATGGTTCTGGAcc                             <  1:419649/301‑1 (MQ=255)`  
                                                                                                                                                                                                                                                                                    `ggTCCGCCGAGTTGACCTCACAGAACATGCAGAAGGTCCAAGATATTGGGTCTATACGAGCGGCTAACGGAGAGAATATGCTGGAAGGTTACACAATGGACCGCAGTAAGCGAGTCACAGAAGGACAGTTCATTCGGGAAGACAATATGGTAACTGAGAACTATCACCGTGACTACCAAGCAATCTTCGCACAGCAACTTGGTGGTACGCAAAGTGCTGCAAGTCAGATTGACGAAATCTATAAGAGCGAACAGAAACAGAAGAGTAAGCTACAGATGGTTCTGGACCCACTGGATATCAt                <  1:140506/300‑1 (MQ=255)`  

                                                                                                                                                                                                                                                                                                     `|`                                                                                                                                                                                                                                                                                                           
`CCTCAAGACTATCGGTGCGGTATTCCATGAAGAGTACACACGAGATGGTCAATTTCAGTTATTTACAATCACGAAAGGAGGATAACCATATGTGTTGGGCAGCCGCAATACCTATCGCTATATCTGGCGCTCAGGCTATCAGTGGTCAGAACGCTCAGGCCAAAATGATTGCCGCTCAGACCGCTGCTGGTCGTCGTCAAGCTATGGAAATCATGAGGCAGACGAACATCCAGAATGCTGACCTATCGTTGCAAGCTCGAAGTAAACTTGAGGAAGCGTCCGCCGAGTTGACCTCACAGAACATGCAGAAGGTCCAAGCTATTGGGTCTATCCGAGCGGCTATCGGAGAGAGTATGCTTGAAGGTTCCTCAATGGACCGCATTAAGCGAGTCACAGAAGGACAGTTCATTCGGGAAGCCAATATGGTAACTGAGAACTATCGCCGTGACTACCAAGCAATCTTCGCACAGCAACTTGGTGGTACTCAAAGTGCTGCAAGTCAGATTGACGAAATCTATAAGAGCGAACAGAAACAGAAGAGTAAGCTACAGATGGTTCTGGACCCACTGGCTATCATGGGGTCTTCCGCTG  >  NC_001604.1/27639‑28229`

  

Alignment Legend

Aligned base mismatch/match (shaded by quality score): `ATCG/ATCG` `< 3 ≤` `ATCG/ATCG` `< 15 ≤` `ATCG/ATCG` `< 27 ≤` `ATCG/ATCG` `< 37 ≤` `ATCG/ATCG` `< 38 ≤` `ATCG/ATCG`

Unaligned base: `atcg`    Masked matching base: `atcg`    Alignment gap: `‑`    Deleted base: `‑`
